# The Impact of Maternal Gestational Diabetes Mellitus on Minipuberty in Boys

**DOI:** 10.3390/nu16234145

**Published:** 2024-11-29

**Authors:** Karolina Kowalcze, Sofia Burgio, Johannes Ott, Giuseppe Gullo, Simona Zaami, Robert Krysiak

**Affiliations:** 1 Department of Pathophysiology, Faculty of Medicine, Academy of Silesia, Rolna 43, 40-555 Katowice, Poland; 2 Department of Pediatrics in Bytom, Faculty of Health Sciences in Katowice, Medical University of Silesia, Stefana Batorego 15, 41-902 Bytom, Poland; 3 Department of Obstetrics and Gynecology, Villa Sofia Cervello Hospital, University of Palermo, 90146 Palermo, Italy; sofiaburgio4@gmail.com (S.B.); gullogiuseppe@libero.it (G.G.); 4 Clinical Division of Gynecologic Endocrinology and Reproductive Medicine, Department of Obstetrics and Gynecology, Medical University of Vienna, 1090 Vienna, Austria; johannes.ott@meduniwien.ac.at; 5 Department of Anatomical, Histological, Forensic and Orthopedic Sciences, Sapienza University of Rome, 00161 Rome, Italy; simona.zaami@uniroma1.it; 6 Department of Internal Medicine and Clinical Pharmacology, Medical University of Silesia, Medyków 18, 40-752 Katowice, Poland; rkrysiak@sum.edu.pl

**Keywords:** genital organs, diabetes complications, hypothalamic–pituitary–testicular axis, hyperglycemia, male infants, saliva

## Abstract

Background/Objectives: Minipuberty is thought to play an important role in the sexual maturation of infants. Maternal disorders during pregnancy were found to have an impact on the activity of the reproductive axis in the first year of life. This prospective, matched, cohort study was aimed at investigating whether the course of minipuberty in boys is affected by maternal gestational diabetes mellitus (GDM). Methods: The study population consisted of three matched groups of boys: infants born to women with poorly controlled GDM, sons of women with adequately controlled GDM, and infants of healthy women with normal carbohydrate tolerance during pregnancy (control group). Salivary levels of testosterone, androstenedione, DHEA-S and estradiol, and urinary concentrations of FSH and LH were repeatedly measured over the first 12 months of life. Hormone levels were correlated with the size of genital organs (testicular volume and penile length), which were measured at each visit. Results: Compared with the remaining groups, the male offspring of women with poorly controlled GDM were characterized by higher concentrations of both gonadotropins, higher salivary testosterone levels, lower salivary DHEA-S concentrations, and longer periods of detection for LH and testosterone. Levels of gonadotropin, testosterone and DHEA-S in sons of mothers with poorly controlled GDM correlated with mean levels of glycated hemoglobin during pregnancy. Moreover, the infant boys assigned to this group were characterized by larger sizes of the testes and penis. Over the entire study period, there were no differences in hormone levels, testicular volume and penile length between sons of adequately treated women with GDM and sons of healthy women. Conclusions: The obtained results indicate that GDM, if poorly controlled, may affect the activity of the reproductive axis and postnatal growth of male genital organs in the offspring.

## 1. Introduction

The term minipuberty refers to the second of the three key phases of reproductive axis activation that occurs after birth (the first phase is observed in the late first and the second trimester of pregnancy, while the third phase, puberty, begins in late childhood or in adolescence) [1,2]. In healthy infant boys, concentrations of follicle-stimulating hormone (FSH), luteinizing hormone (LH) and testosterone start to increase after the first week of age, reaching peak values between 4 and 12 weeks of life, and dropping to prepubertal levels between 6 and 9 months of age (FSH and LH) or at around 6 months of life (testosterone) [1,3]. Compared to infant girls, infant boys are characterized by higher concentrations of LH, lower concentrations of FSH and shorter periods of detection for FSH and sex hormones [4,5]. The physiological significance of minipuberty in boys is better understood than in girls. Reproductive axis activation in this period of life is associated with growth of the testes and external male genitalia (especially the penis), as well as with changes in the cellular composition of the testes (particularly with an increase in the number of the immature Sertoli cells and with a transient increase in the number of Leydig cells) [3]. Increased testosterone production during minipuberty seems to be a consequence of increased LH secretion, and is probably mediated through the LH receptor, which is, already during fetal life, localized on the cellular membrane of Leydig cells [2]. In turn, activation of the FSH receptor on the surface of Sertoli cells results in quantitative and morphological changes in these cells [4]. Except for a small increase in testicular size and penile enlargement, activation of the reproductive axis is not followed by clinically visible physical changes in infant boys [1]. Moreover, minipuberty is postulated to influence body composition and linear growth, contributing to accumulation of more lean mass and a higher growth velocity in males than females [4,5]. Lastly, minipuberty is speculated to play a role in forming brain plasticity, and may be implicated in sensory and cognitive maturation [6].

Gestational diabetes mellitus (GDM), defined as hyperglycemia of any degree that begins or is first recognized during pregnancy, is the most prevalent disorder in this period of life (up to 15–25% of all pregnancies) [7,8]. The disorder impairs the health of several million women worldwide, and is associated with increased morbidity and mortality in the offspring [9]. In addition to perinatal complications, including macrosomia, hypoglycemia, prematurity, birth injury, respiratory distress syndrome, polycythemia, hyperbilirubinemia and hypocalcemia, chronic exposure to maternal GDM in utero plays a causative role in determining long-term adverse offspring outcomes: adiposity, cardiometabolic diseases and probably also lower cognitive ability [10,11,12]. Most of these complications can be prevented by proper treatment during pregnancy [13].

Although GDM is one of the most common health problems affecting pregnant women, no study has investigated its association with minipuberty. In addition, only a few studies have assessed the impact of this disorder on male puberty. In the British Avon Longitudinal Study of Parents and Children cohort study, sons of mothers with GDM attained a pubic hair Tanner stage higher than 2 at a slightly younger age (two months earlier) than boys born to women without this disorder [14]. Grunnet et al. reported higher percentages of boys with testicular size exceeding 4 mL and with pubic hair Tanner stage 2 or higher if they were the offspring of mothers with GDM [15]. Lauridsen et al. did not observe significant differences in the mean age at achieving the pubertal milestones between sons prenatally exposed to maternal GDM (or preexisting type 1 and type 2 diabetes) compared with sons born to mothers without diabetes [16]. Davis et al. observed no differences in Tanner stages among offspring of mothers with GDM and control offspring, but their study analyzed boys together with girls [17]. Lastly, the median age of pubertal timing after adjustment for the child’s sex and race/ethnicity in the Early Perinatal Outcomes in Children study was younger in the offspring of women with GDM, as well as that exposed males were found to have a 4.0% greater peak height velocity compared with unexposed ones [18]. Unlike maternal diabetes, diabetes in children caused delayed puberty. The delay depended on the disease duration and treatment effectiveness, and was slight or even absent in boys achieving therapeutic goals [19,20].

Recent studies of our research team showed that the course of male minipuberty was affected by other chronic maternal disorders: hypothyroidism [21] and hypovitaminosis D [22], but their impact was completely different. Sons of women with hypothyroidism uncontrolled or poorly controlled during pregnancy were characterized by decreased activity of the reproductive axis [21]. In turn, low maternal vitamin D status during pregnancy predisposed the male offspring to increased activity of this axis, correlating with severity of deficiency [22]. There are also studies showing changes in male minipuberty in cases of prematurity and abnormal birth weight. Prematurity, one of the complications of GDM [10,11,12], was characterized by higher levels of testosterone and LH, as well as by faster postnatal growth of genital organs (the testes and penis) [23]. In turn, infant boys born small for their gestational age differed from those born appropriate for their gestational age in LH and FSH concentrations [24,25], and it is possible that increased body weight (a typical complication of GDM) may affect the activity of the reproductive axis. Given the high prevalence of GDM, the lack of dedicated studies, and theoretical premises, the aim of this study was to investigate the association between GDM and male minipuberty. Unlike the Tanner staging of puberty, there is no dedicated scale for evaluating the initiation and progression of minipuberty. Thus, the clinical follow-up included penile growth velocity and changes in testicular volume, which are indirect markers of, respectively, secretory function of Leydig cells and pituitary gonadotropes [2,5]. In addition, we determined, also for the first time, androgen production by the adrenal glands in male infants of mothers with GDM.

## 2. Materials and Methods

The study was carried out between July 2022 and September 2024. The study protocol was approved by the institutional committee on human research (the Bioethical Committee of the Medical University of Silesia-PCN/CBN/0052/KB1/17/22; 12 April 2022), and all study procedures were performed in accordance with the ethical guidelines and principles of the Declaration of Helsinki. A parent or legal guardian provided written informed consent for their child’s participation after a comprehensive discussion about the research objectives, methods, and potential risks associated with participation. Due to its purely observational character, the study did not require registration at a clinical trial registry.

### 2.1. Participants

**Figure 1:**
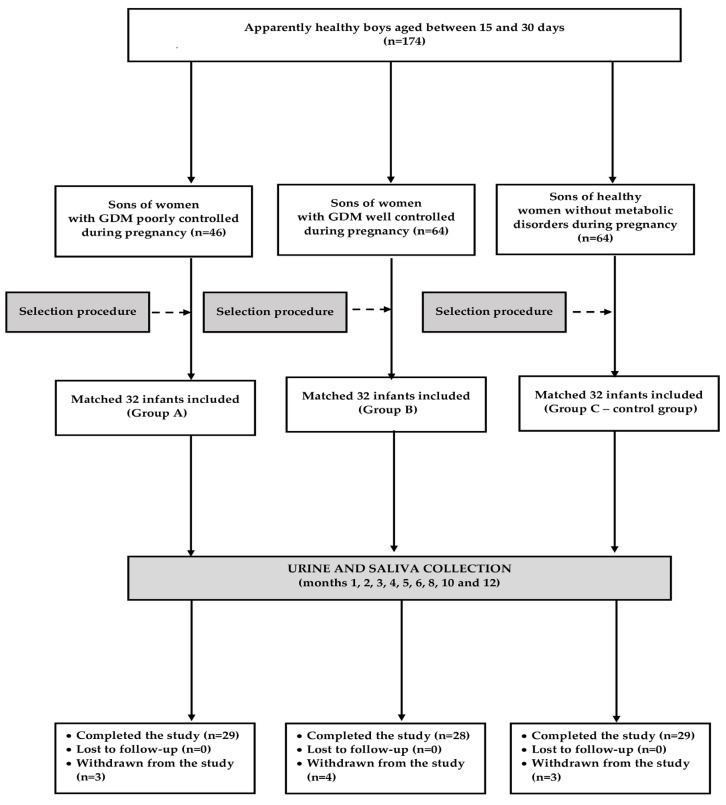
The flow of the participants through the study.

The participants of this prospective cohort study were recruited among 174 apparently healthy male neonates aged between 15 and 30 days. Based on maternal glucose homeostasis in pregnancy, the infants were assigned to one of three groups: boys born to women with poorly controlled GDM (group A), sons of women with GDM meeting glycemic targets (group B), and male descendants of women with normal glucose homeostasis during pregnancy (group C—the control group). GDM was diagnosed at any time in pregnancy if one or more of the following criteria were met: fasting plasma glucose 5.1–6.9 mmol/L (92–125 mg/dL), 1 h plasma glucose ≥ 10.0 mmol/L (180 mg/dL) following a 75 g oral glucose load; 2 h plasma glucose 8.5–11.0 mmol/L (153–199 mg/dL) following a 75 g oral glucose load [7]. The 75 g oral glucose tolerance test was performed in all mothers. Women with glucose concentrations below these values and, if performed, with HbA_1c_ < 5.7% (39 mmol/mol) were considered to have normal glucose homeostasis. Group B included women with mean fasting plasma glucose during treatment < 5.3 mmol/L (95 mg/dL), mean 1 h postprandial glucose < 7.8 mmol/L (140 mg/dL), mean 2 h postprandial glucose < 6.7 mmol/L (120 mg/dL), and mean glycated hemoglobin (HbA_1c_) below 6.0% (42 mmol/mol). If the mean levels of fasting glucose, postprandial glucose and HbA_1c_ exceeded these values, GDM was considered poorly controlled, and the infant was assigned to group A. During pregnancy, all women with GDM were asked to comply with dietary recommendations, and some received insulin therapy. Daily calorie requirements depended on the recommended rate of weight gain: 0.44–0.58 kg/week for underweight women (pre-pregnancy body mass index [BMI] < 18.5 kg/m^2^), 0.35–0.50 kg/week for normal weight women (pre-pregnancy BMI between 18.5 and 24.9 kg/m^2^), 0.23–0.32 kg/week for overweight women (pre-pregnancy BMI between 25.0 and 29.9 kg/m^2^), and 0.17–0.27 kg/week for obese women (pre-pregnancy BMI ≥ 30.0 kg/m^2^). Carbohydrate intake was recommended to be restricted to 50% of total calories, with a minimum of 175 g per day. The recommended daily allowances for protein and fiber were 71 g and 28 g, respectively. Before the study began, a sample size calculation was performed in order to establish the number of participants that would be required to detect a 20% difference in the primary endpoint (between-group difference in salivary estradiol levels) with a power (β) of 0.8 and a confidence (α) of 0.05. The sample size was estimated at 25 infants per group. However, to account for the potential loss of the participants, this number was increased by more than a quarter, and 32 infants were included in each group. The participants were selected from a larger cohort of infant boys meeting the inclusion and exclusion criteria (Figure 1). This selection was aimed at obtaining three groups matched for maternal age, maternal BMI, gestational age of delivery, birth order, and a family history of diabetes in pregnancy. The matching algorithm was based on the minimum Euclidean distance rule [26]. All necessary data were extracted from medical records of mothers and infants. To minimize seasonal confounds and fluctuations in hypothalamic–pituitary–gonadal axis activity, similar numbers of boys were recruited between December and February (9 in group A, 7 in group B and 9 in group C), between March and May (7 in group A, 9 in group B and 8 in group C), between June and August (8 in each group), and between September and November (8 in groups A and B, 7 in group C).

Potential participants were not considered for enrollment if the mothers had preexisting type 1 or type 2 diabetes, overt diabetes in pregnancy or documented preexisting prediabetes; if mothers had discordant glucose or HbA_1c_ values; if mothers were diagnosed with other chronic diseases; if mothers had any complications requiring urgent hospital admission during pregnancy, or were addicted to drugs or alcohol; or if mothers were treated for a period exceeding 10 days (except for insulin and vitamin/micronutrient supplements for pregnant and breastfeeding women). Glucose or HbA_1c_ values were considered discordant if some results suggested poor disease control, while the remaining results suggested adequate disease control. Potential participants were also excluded if they had genetic syndromes, major congenital anomalies, cryptorchidism, congenital infections, birth asphyxia, any chronic disorder, were born before week 36 of pregnancy, or were chronically treated (except for vitamin D supplementation).

### 2.2. Study Design

The infant boys were followed-up until the end of the first year: once a month in the first 6 months of life, and every two months thereafter. At each visit, the parent or guardian was asked about the boy’s health status and medication usage since the last visit. Moreover, the investigator thoroughly examined the infant, and analyzed the results of the performed laboratory and imaging tests. Urine and saliva samples were collected only if the infant was considered healthy, and had not been treated with any medications (except for vitamin D supplementation and obligatory vaccinations) in the last 10 days. The infants were considered eligible for the final analysis only if urine and saliva were collected at least seven times (at least five times in the first six months of life, and at least twice between month 6 and month 12). The flow of patients through the study is shown in Figure 1.

Consumption of nutrients during pregnancy was calculated at the first visit. For mothers with GDM, these calculations were based on the analysis of dietary diaries completed during pregnancy. In turn, women with normal glucose homeostasis during pregnancy were asked to complete a questionnaire evaluating what amount and how often in the last eight weeks of pregnancy (daily, 5–6 times per week, 3–4 times a week, 1–2 times a week, less frequently or never) they had consumed each of the most common meals in Polish cuisine. The obtained data and food composition and nutrition tables were used to calculate the mean daily intake of calories, carbohydrates proteins, lipids, fibers and vitamin D.

Infant weight, length and head circumference were measured during each visit using a calibrated scale or a portable infantometer (SECA, Hamburg, Germany). Nude weight was measured to the nearest 10 g, while length and head circumference were measured to the nearest 1 mm. Length was measured from the crown of the head to the foot’s heel while both legs were stretched simultaneously and the chin supported perpendicular to the surface. Head circumference was measured from the glabella to the posterior external protuberance using a flexible measuring tape with 1 mm accuracy. Weight-for-length was calculated by dividing the infant’s weight by length, and the obtained quotient was converted to a percentile score using World Health Organization gender-specific growth charts [27]. BMI was calculated by dividing the mother’s mass by her height squared.

Testicular volume and penile length were measured by the principle investigator three times, and the results were averaged. Testicular volume was measured using a high-resolution, small-part transducer (Esaote MyLab Six), operating at frequencies ranging from 5 MHz to 12 MHz. The left testis was measured first, followed by the right testis. According to our previous experience, this measurement order was associated with the best intra-rater reproducibility. Separate transverse and longitudinal images of each testis were obtained. The longitudinal diameter (length) in the maximum longitudinal scan, anterior–posterior diameter (width) in the maximum longitudinal scan, and the lateral–medial diameters (thickness) in the maximum transverse scan were measured and recorded. The volume of each testis was calculated using the empirical formula of Lambert (length × width × thickness × 0.71), displaying a strong linear relationship with the true volume and providing better accuracy than other formulas [28]. The epididymis was excluded from the measurements. The sum of the volumes of both testes was then divided by 2 to calculate the mean testicular volume. Penile length was measured using a calibrated tape. Measurements were taken by gently extending the penis to its maximum length without causing pain. The length was measured from the pubic ramus to the tip of phallus glans excluding the prepuce.

### 2.3. Laboratory Assays

Due to diurnal variations [29], samples of saliva and urine were collected between 7.30 and 9.00 a.m. in a quiet and air-conditioned room (constant temperature of 23–24 °C). The blunt tips of 2 mL sterile syringes were placed in the oral cavity, and saliva samples were collected by gentle vacuum aspiration. The whole procedure lasted on average 40 ± 10 s, was painless and did not induce stress in the participants. To prevent food debris and other contaminants from affecting sample accuracy, the participants were not fed for at least 1 h before the procedure. The collected saliva was then centrifuged to remove residual material or oral mucous cells, and the upper layer was transferred to polypropylene tubes. Urine samples were collected using sterile pediatric urine collection bags (Medicavera, Szczecin, Poland). Care was taken not to collect the first morning urine. After thorough cleansing the genital area and allowing the skin to air dry, the bag was firmly attached to the child’s genital area in order to minimize leakage and contamination during the collection procedure. Saliva and urine samples were then stored at −20 °C temperature and prior to analysis, they were thawed at room temperature.

The assays were carried out in duplicate to ensure accuracy, and the results were averaged. Urine levels of FSH and LH were measured by a solid-phase, two-site chemiluminescent immunometric assay (Siemens Healthcare Diagnostics, Erlangen, Germany). The obtained measurements were adjusted for creatinine (determined using a routine technique [Roche Diagnostics, Basel, Switzerland]) in order to address variability in the dilution of urine. Salivary levels of testosterone, androstenedione, dehydroepiandrosterone sulfate (DHEA-S) and estradiol were measured using enzyme-linked immunosorbent assays [21,22]. The minimum detectable levels (limits of detection—LOD) for the assessed hormones were 10 pmol/L (testosterone), 18 pmol/L (androstenedione), 100 nmol/L (DHEA-S), 4 pmol/L (estradiol), 0.1 U/L (FSH) and 0.1 U/L (LH).

### 2.4. Statistical Analysis

All continuous variables were logarithmically transformed to correct for non-normality, to stabilize variance and to reduce the impact of extreme values (outliers). An LOD value was assigned for undetectable levels if the other concentrations were above the LOD. Data normality and homogeneity of variances were tested using Shapiro–Wilk’s and Levene’s test, respectively. Because the log transformed variables met the assumptions of both normality and homoscedasticity (the prerequisites of analysis of variance), inter-group comparisons at the same time point were carried out using one-way analysis of variance followed by Bonferroni’s test. Within-group comparisons at different time points were carried out using repeated measures analysis of variance, followed by Tukey’s post hoc test for pairwise comparisons. Categorical variables were compared using the chi-square test. Bivariate relationships were assessed using Pearson’s r tests (for two continuous variables); phi coefficient (for one continuous and one categorical variable); and point-biserial (for two categorical variables). Intra-rater reliability was assessed using intraclass correlation coefficients. Two-tailed *p*-values corrected for multiple testing less than 0.05 were considered statistically significant.

## 3. Results

Ten boys prematurely terminated the study. Recurrent respiratory infections did not allow us to obtain the required minimum numbers of samples in five infants (two from groups A and C, and one from group B). Two patients (assigned to groups A and C) developed atopic dermatitis and gastroesophageal reflux, and required chronic pharmacotherapy. The parents of one boy (assigned to group B) withdrew consent without giving a reason. Two infants (from group B) were withdrawn because of misdiagnosis and treatment during pregnancy. Twelve weeks after delivery, one of them was diagnosed with type 2 diabetes (likely present already during pregnancy), while another one admitted that she had been treated with methyldopa (which was not mentioned in her medical record). The remaining 86 infants (89.6%) completed the study, and were included in the final analyses. The post hoc power analysis showed that the final sample size was sufficient to achieve the purposes of the study. The intraclass correlation coefficient was 0.93 ± 0.04 for testicular volume and 0.95 ± 0.02 for penile length, indicating the high reliability of the measurements.

Similar proportions of mothers with GDM adhered to dietary recommendations (group A: 82.8%, group B: 85.7%), and received insulin (group A: 20.6%, group B: 21.4%). The cumulative insulin dose was similar in both these groups (1706 ± 542 units vs. 1884 ± 592 units, *p* = 0.5988). No woman was treated with any other hypoglycemic agents. The mothers did not differ with regard to age, education, occupational activity, employment, socioeconomic status, smoking, BMI, systolic blood pressure, diastolic blood pressure, the mean daily intake of calories, carbohydrates, lipids, proteins and fibers, the mean daily vitamin D intake (with food and supplements), and their family history of diabetes during pregnancy. The mean fasting glucose concentration during pregnancy was the highest in mothers of infants from group A, but similar in mothers of infants assigned to the remaining groups (Table 1). Groups A and B differed in the mean levels of postprandial glucose and HbA_1c_ during pregnancy, which were higher in group A (1 h postprandial glucose: 9.11 ± 0.94 mmol/L vs. 7.00 ± 0.61 mmol/L, *p* < 0.0001; 2 h postprandial glucose: 7.56 ± 0.78 mmol/L vs. 5.72 ± 0.56 mmol/L, *p* < 0.0001; HbA_1c_: 6.3 ± 0.2% vs. 5.2 ± 0.3%, *p* < 0.0001).

The study groups did not differ in gestational age of delivery, birth order, body length, head circumference, breastfeeding or total daily (with food and supplements) intake of vitamin D. Compared with groups B and C, group A was characterized by higher weight and higher weight-for-length percentile (Table 2).

**Figure 2:**
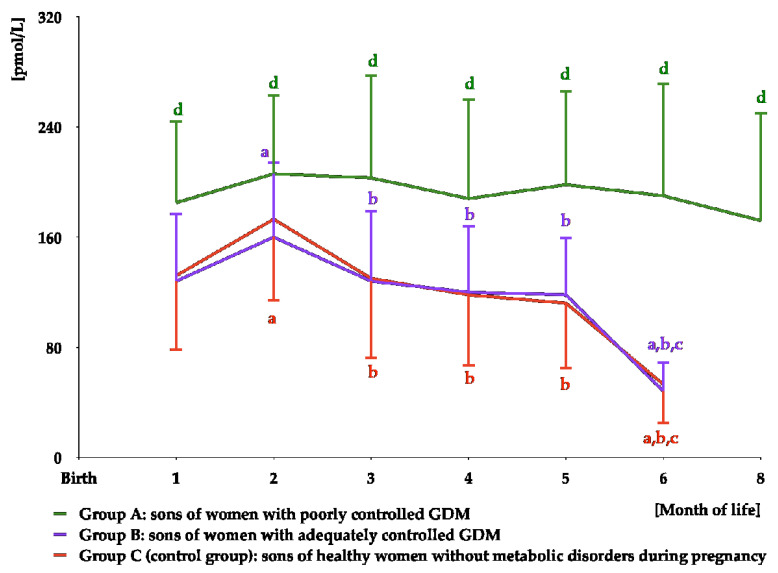
Salivary testosterone concentrations in infant boys participating in the study. Testosterone was undetectable in saliva from month 10 to month 12 in group A, and from month 8 to month 12 in groups B and C. In statistical comparisons, the limit of detection (LOD) value was assigned for testosterone in groups B and C at month 8. ^a^ *p* < 0.05 vs. levels at month 1 in the same study group; ^b^ *p* < 0.05 vs. levels at month 2 in the same study group; ^c^ *p* < 0.05 vs. levels at months 3–5 in the same study group; ^d^ *p* < 0.05 vs. levels in groups B and C at the same time point.

In group A, testosterone was detectable in saliva for the first eight months of life, while in groups B and C for the first six months of life. In group A, over the entire period of detection, testosterone levels were stable. In the remaining groups, the highest concentrations were observed at month 2, and decreased thereafter. Over the entire period of detection, testosterone concentrations were higher in group A than in the remaining groups, but did not differ between groups B and C (Figure 2 and Supplementary Table S1). The area under the curve for testosterone was greater in group A than in groups B and C (Table 3).

**Figure 3:**
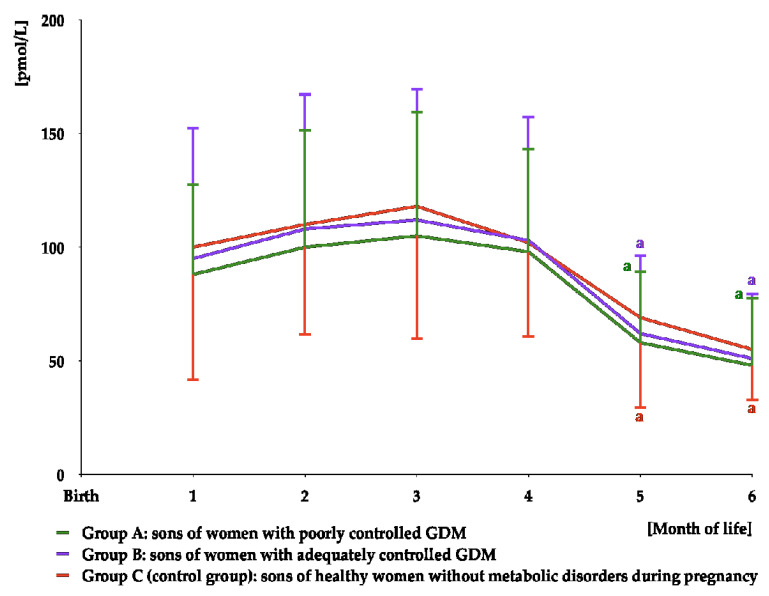
Salivary androstenedione concentrations in infant boys participating in the study. Androstenedione was undetectable in saliva from month 8 to month 12. ^a^ *p* < 0.05 vs. levels during the first 6 months of life.

In all study groups, androstenedione was detectable in saliva from month 1 to month 6. Stable concentrations of this hormone were observed between months 1 and 4, and decreased thereafter. There were no differences in androstenedione levels between the study groups (Figure 3 and Supplementary Table S2). The area under the curve for androstenedione did not differ between the groups (Table 3).

**Figure 4:**
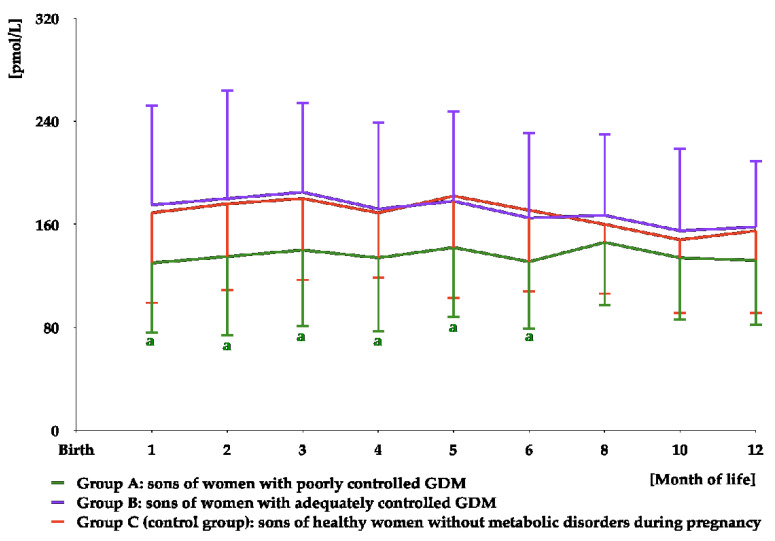
Salivary DHEA-S concentrations in infant boys participating in the study. ^a^ *p* < 0.05 vs. levels in groups B and C at the same time point.

DHEA-S was detectable in saliva over the entire study period. For the first six months of life, its concentrations were lower in group A than in the remaining two groups, but did not differ between group B and group C. There were no differences in DHEA-S levels at different time points in the same group (Figure 4 and Supplementary Table S3). The area under the curve for DHEA-S was slightly lower in group A than in groups B and C (Table 3).

**Figure 5:**
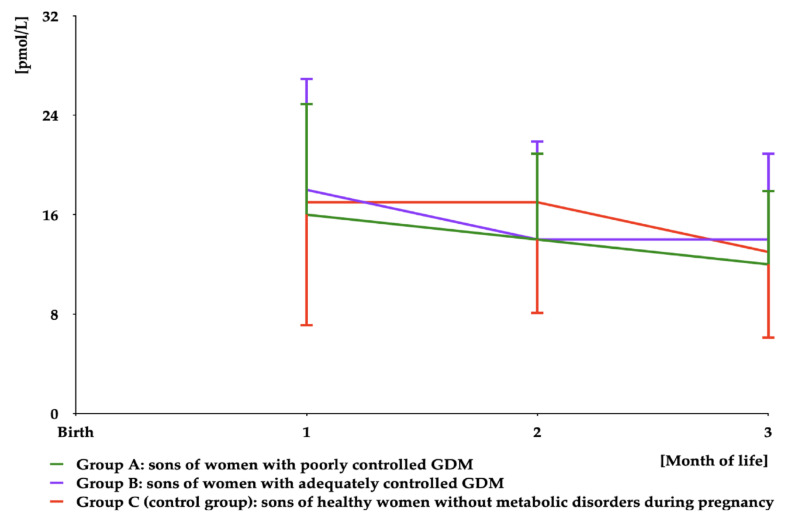
Salivary estradiol concentrations in infant boys participating in the study. Estradiol was undetectable in saliva from month 4 to month 12.

Estradiol was detectable in saliva during the first three months of life. There were no differences between the groups in levels of this hormone (Figure 5 and Supplementary Table S4). The area under the curve for estradiol was similar in all study groups (Table 3).

**Figure 6:**
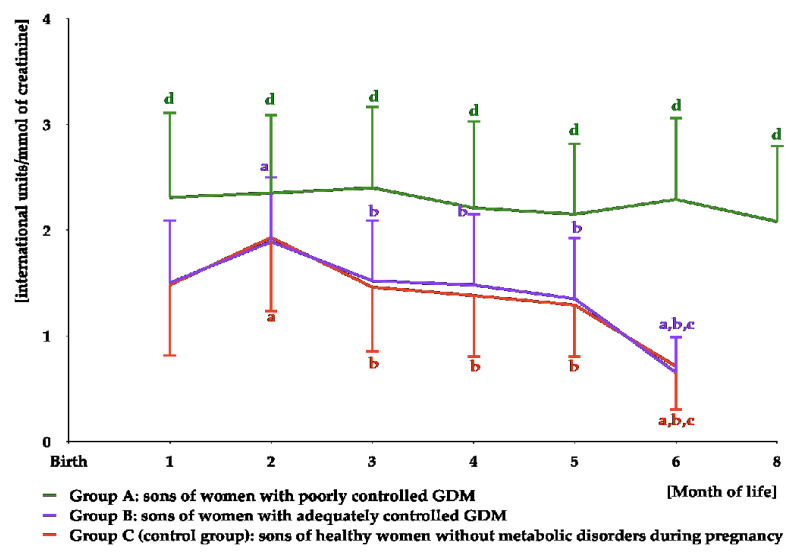
Urinary LH levels in infant boys participating in the study. LH was undetectable in saliva from month 10 to month 12 in group A, and from month 8 to month 12 in groups B and C. In statistical comparisons, the limit of detection (LOD) value was assigned for LH in groups B and C at month 8. ^a^ *p* < 0.05 vs. levels at month 1 in the same study group; ^b^ *p* < 0.05 vs. levels at month 2 in the same study group; ^c^ *p* < 0.05 vs. levels at months 3–5 in the same study group; ^d^ *p* < 0.05 vs. levels in groups B and C at the same time point.

In group A, LH was detectable in urine for the first eight months of life, while in groups B and C for the first six months of life. In group A, LH concentrations remained stable over the entire period of detection, while in the remaining groups, the concentrations were the highest at month 2 and decreased thereafter. Over the entire period of detection, LH levels were higher in group A than in the remaining groups, but did not differ between groups B and C (Figure 6 and Supplementary Table S5). The area under the curve for LH was greater in group A than in groups B and C (Table 3).

**Figure 7:**
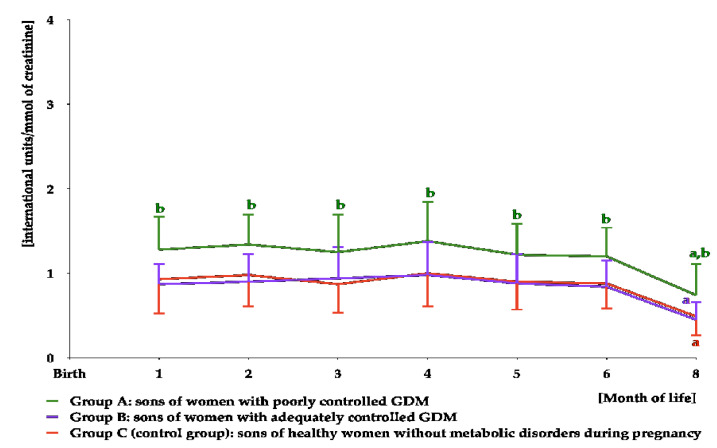
Urinary FSH levels in infant boys participating in the study. FSH was undetectable in urine from month 10 to month 12. ^a^ *p* < 0.05 vs. levels during the first 6 months of life; ^b^ *p* < 0.05 vs. levels in groups B and C at the same time point.

FSH was detectable in urine samples for the first 8 months of life. The levels were stable between months 1 and 6, and decreased thereafter. Over the entire detection period, FSH concentrations were higher in group A than in the remaining groups, but did not differ between groups B and C (Figure 7 and Supplementary Table S6). The area under the curve for FSH was greater in group A than in the remaining study groups (Table 3).

**Figure 8:**
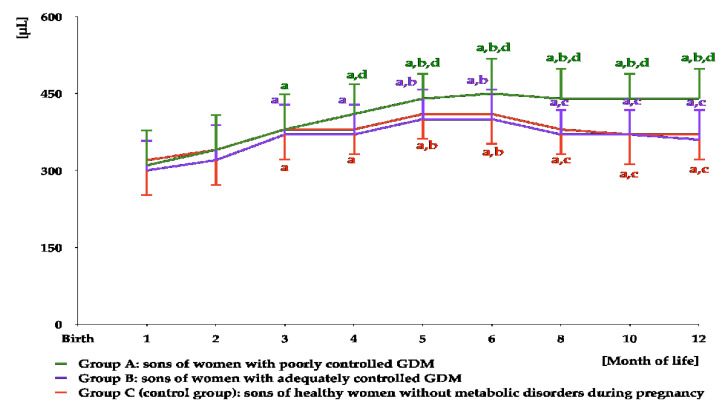
Testicular volume in infant boys participating in the study. ^a^ *p* < 0.05 vs. values at months 1–2 in the same study group; ^b^ *p* < 0.05 vs. values at months 3–4 in the same study group; ^c^ *p* < 0.05 vs. values at months 5–6 in the same study group; ^d^ *p* < 0.05 vs. values in groups B and C at the same time point.

In all study groups, testicular volume increased from month 1 to months 5–6. From month 6 to month 12, there was a significant decrease in this parameter in groups B and C, but not in group A. From month 4 to month 12, testicular volume was greater in group A than in the remaining groups, but did not differ between groups B and C (Figure 8 and Supplementary Table S7). The area under the curve for testicular volume was greater in group A than in groups B and C (Table 3).

**Figure 9:**
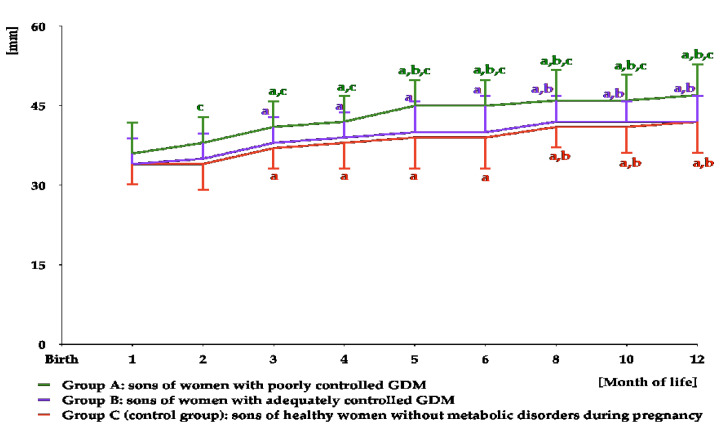
Penile length in infant boys participating in the study. ^a^ *p* < 0.05 vs. values at months 1–2 in the same study group; ^b^ *p* < 0.05 vs. values at months 3–4 in the same study group; ^c^ *p* < 0.05 vs. values in groups B and C at the same time point.

During the study, penile length increased with time. From postnatal month 2 to the end of the follow-up, the length was greater in group 1 than in the remaining two groups. No differences in penile length were observed between groups A and B (Figure 9 and Supplementary Table S8). The area under the curve for penile length was greater in group A than in groups B and C (Table 3).

In all study groups, over the entire period of detection, there were positive correlations between urinary LH levels and salivary testosterone concentrations, between urinary FSH concentrations and testicular volume, and between salivary testosterone levels and penile length. In group A, but not in group B, there were also positive correlations between mean maternal HbA_1c_ levels and concentrations of FSH, LH and testosterone, and inverse correlations between mean maternal HbA_1c_ levels and DHEA-S concentrations. Table 4 shows correlations between the assessed variables. Correlations between the outcome measures and the daily intake of energy, macronutrients and vitamin D, as well as the remaining correlations were insignificant. All correlations persisted after adjustment for gestational age of delivery, and for infants’ body weight and weight-for-length percentile.

## 4. Discussion

The major finding of this study is that poorly controlled GDM leads to increased activity of the hypothalamic–pituitary–testicular axis during infancy. Boys born to mothers with poorly controlled GDM were characterized by higher concentrations of gonadotropins and testosterone, longer periods of detection for LH and testosterone, larger areas under the curves for gonadotropins and testosterone, and by the lack of clear peaks in levels of LH and testosterone, which were observed in the remaining groups. Moreover, the degree of elevation in gonadotropin and testosterone levels in the former group was determined by mean levels of HbA_1c_, the main biomarker assessing long-term glycemic control, despite the fact that because of enhanced red blood cell turnover, these levels are slightly lower during pregnancy [30]. Moderate and strong positive correlations between urinary LH concentrations and salivary testosterone levels, and the same periods of their detection suggest that increased hormonal activity of the testes is likely to be secondary to the impact of disturbances in glucose homeostasis at the level of the pituitary gonadotropic cells and/or hypothalamic secretion of the gonadotropin-releasing hormone, the main regulator of LH and FSH synthesis and release [31].

The most important covariates that might have theoretically affected our findings were differences in adipose tissue content and prematurity [32]. To minimize their impact, the study groups were matched for maternal BMI and gestational age of delivery, while birth before week 36 of pregnancy excluded potential participants from the study. Despite matching, infants of mothers with poorly controlled GDM had a higher body mass and a higher weight-for-length percentile, probably resulting from enhanced secretion of insulin by the fetal pancreas in response to hyperglycemia [32]. However, both parameters did not correlate at any time point with hormone levels (and with testicular volume and penile length), which argues against their role in determining between-group differences in the course of minipuberty. This explanation is also in line with the finding that boys with Prader–Willi syndrome were characterized by FSH, LH and testosterone levels within the normal minipubertal range, though this syndrome is considered the most common genetic cause of morbid obesity in children [33,34]. Lastly, our findings cannot be attributed to differences in maternal vitamin D status, despite the impact of low vitamin D status on the course of male minipuberty [22] and a significant association between vitamin D deficiency and increased risk of GDM [35]. The daily intake of this vitamin by the mothers was similar in all study groups and high enough to ensure normal vitamin D status [36].

Positive correlations between LH and testosterone suggest that the highest salivary testosterone levels in sons of mothers with poorly controlled GDM should be attributed to the most pronounced secretory function of intestinal cells of Leydig in this group of boys. Testosterone detectable in saliva during the first eight months of life and between-group differences in this parameter do not seem to be a consequence of androgen production by the adrenal glands and/or differences in the conversion rate to testosterone. At the same time, DHEA-S concentrations were consistently lower in the poorly controlled group, and did not correlate with testosterone levels. This finding and the lack of correlations between urinary gonadotropins and salivary DHEA-S suggest that impaired adrenal androgen production in sons of mothers with poorly controlled GDM is not related to activation of the hypothalamic–pituitary–testicular axis. Furthermore, higher testosterone levels in sons of mothers with poorly controlled GDM cannot be explained by differences in transplacental transport and/or in maternal blood levels during pregnancy. To eliminate the impact of maternal sex hormones, which are known to penetrate the placental barrier [37], the study did not include infants younger than two weeks of age. Between 15 and 30 days of life (the age at which all boys started participation in the study), maternal sex hormones were almost certainly absent in the infant’s saliva due to their short half-lives (10 min for testosterone and 20–30 min for estradiol) [38,39].

Increased activity of the hypothalamic–pituitary–testicular axis in descendants of women with poorly controlled GDM is likely to contribute to greater dimensions of the genital organs, observed since month 2 (penile length) and since month 4 (testicular volume) until the end of the follow-up period. In line with this explanation, testicular volume positively correlated with urinary FSH concentrations, and similar correlations were observed between penile length and salivary testosterone concentrations. These correlations persisted after adjustment for body weight and weight-for-length percentile (differing between sons of women with poorly controlled GDM and the remaining study groups), as well as after adjustment for gestational age of delivery. Positive correlations between FSH and testicular volume may be explained by the fact that FSH signaling plays a pivotal role in regulating growth and function of Sertoli cells (even immature), and in increasing spermatogonia number [40]. The weaker strength of correlations between FSH and testicular volume in sons of mothers with poorly controlled GDM than in the remaining groups suggests an additional, FSH-unrelated effects of hyperglycemia on Sertoli cells and/or spermatogonia, or on autocrine or paracrine regulators of these cells. This interpretation may also explain why sons of women with poorly controlled disease were characterized by no changes in testicular volume after month 6, though the volume decreased in the remaining groups, and a similar decrease in the second half of the first year of life was previously reported in healthy infant boys participating in other studies [23,41]. Interestingly, though there are no data for the offspring of women with elevated glucose levels during pregnancy, as many as one-third of diabetic Greek boys younger than 10 years old were characterized by testicular volume exceeding 3 mL [42]. Thus, it is possible that differences in the dimensions of the testes and/or penis persist over the period of physiological quiescence of the reproductive axis, and, considering the results of animal studies [43], may have an impact on their further development during puberty and on the final size in adulthood. The lack of correlations between salivary testosterone concentration and testicular volume is probably a consequence of the lack of the androgen receptor in Sertoli cells during the first 12 months of life. The resultant androgen insensitivity of these cells protects against their precocious maturation [44].

Differences in testicular volume and penile length cannot be explained by changes in estradiol production. Although estrogen receptors are present in the testis and penis, and while estradiol was found to decrease testicular size and impede normal penile development [45], no between-group differences in salivary estradiol levels, the short detection period (three months) and the lack of correlations with dimensions of the testes and penis indicate that this hormone does not determine testicular and penile growth in infancy, and did not contribute to between-group differences in the size of male genital organs observed in our study.

Comparing the course of minipuberty in sons of women with GDM and in boys born to mothers with other chronic disorders, it seems that different maternal disorders induce specific changes in the reproductive axis activity. Male descendants of mothers with untreated or undertreated hypothyroidism were characterized by lower concentrations of gonadotropins and testosterone during the first 6 months of life, and by later peak levels of LH and testosterone [21]. The opposite changes (lower concentrations of FSH, LH and testosterone, earlier peak concentrations of LH and testosterone) were in turn observed in sons of women with vitamin D deficiency, while vitamin D insufficiency was associated with only slightly elevated FSH concentrations [22]. Although at first glance, the impact of vitamin D deficiency and GDM is similar, there are some differences between them in LH and androgen secretion. Only in the offspring of women with poorly controlled GDM, the detection periods for LH and testosterone were longer than in infant boys born to healthy mothers, and increased testosterone levels were accompanied by lower salivary levels of DHEA-S. In turn, sons of mothers with vitamin D deficiency, but not male descendants of women with poorly controlled GDM, were characterized by the presence of peak concentrations of LH and testosterone. Thus, it may be assumed that there is no one common type of response of the hypothalamic–pituitary–testicular axis to various conditions disturbing fetal homeostasis. In sons of women with poorly controlled GDM, increased activity of the reproductive axis was probably caused by exposure to chronically elevated levels of glucose, which is transferred to the fetus by facilitated diffusion [46]. In line with this explanation, concentrations of FSH, LH and testosterone (and DHEA-S) correlated with elevated levels of HbA_1c_. There are two possible explanations for this finding. According to the first explanation, fetal hyperglycemia triggers overactivity of the reproductive axis in utero, secondarily increasing sensitivity of the reproductive axis to its stimulators in infancy. This explanation is supported by the fact that insulin, produced in increased amounts in response to maternal GDM [8], is a strong stimulator of gonadotropin secretion by pituitary cells [47]. However, fetal exposure to hyperglycemia in GDM is characteristic for the late second and third trimester [8,9,10], occurring later than the intrauterine phase of reproductive axis activation, which makes this explanation less likely. According to the second explanation, increased activity of the hypothalamic–pituitary–testicular axis in the offspring of mothers with poorly controlled disease reflects the impact of nutritional and environmental signals during fetal development, which play a seminal role in determining health trajectories in later periods of life (prenatal or fetal programming) [48]. GDM was found to trigger epigenetic changes that can persist after normalizing glucose availability, resulting in a ‘metabolic memory’ of previous hyperglycemia. Some of these changes, particularly DNA methylation and histone modifications, partially explain later susceptibility to type 2 diabetes, obesity, coronary artery disease, and hypertension [49]. ‘Metabolic memory’ may also stimulate activity of the male reproductive axis in infancy at the level of the pituitary or hypothalamus. In agreement with this explanation, descendants of mothers with GDM were characterized by decreased methylation of the gene for leptin. This hypomethylation is associated with increased production of this adipokine, which is an important activator of gonadotropin secretion and a trigger of puberty onset [50,51]. Other genes that regulate pituitary/hypothalamic ontogeny and function and are susceptible to epigenetic modifications in children of women with GDM include *GNAS* and *IGF-2* [52,53]. Unfortunately, despite the association with GDM and gonadotropin secretion [54], insulin-like growth factors (particularly IGF-1) were not assessed in our study because their measurements in urine require acidification, while IGF-1 (and probably also other insulin-like growth factors) detectable in saliva is locally produced, and the salivary concentrations do not correlate with plasma levels [55].

An additional observation of interest included the normal hormone levels and unaltered growth rates of genital organs in sons of mothers achieving the glycemic targets recommended by the American Diabetes Association [56]. Differences in activity of the hypothalamic–pituitary–testicular axis, testicular volume and penile length between the offspring of mothers with poorly and adequately controlled GDM contrasted with similar proportions of women adhering to dietary recommendations and receiving insulin, and with no differences in the cumulative insulin dose during pregnancy. Thus, it seems that the normal course of minipuberty was a consequence of adequate metabolic control, and was not associated with the impact of the diet itself, or with the direct action of exogenous insulin on the reproductive axis. In line with this explanation, there were no differences in testicular size between prepubertal boys born to women who had participated in a randomized controlled trial comparing insulin and metformin in the treatment of GDM [57]. Thus, our findings argue that GDM-induced changes in the course of minipuberty are preventable if the disorder is effectively managed, and this conclusion supports previous observations concerning this period of life in descendants of women with hypothyroidism during pregnancy [21,58]. They also suggest that the recommended threshold levels for fasting and postprandial glucose are fully justified, and that HBA_1c_ concentrations in women with this disorder should not exceed 6.0%, which is considered the optimal target for pregnant women with pre-existing diabetes [56]. Although reproductive axis activity and sizes of the testes and penis are likely determined by disease control, they do not seem to reflect the total calorie intake and specific effects of individual macronutrients in the late second and third trimester of pregnancy. During the management period, their consumption was similar in both groups of women with GDM, did not differ from that of healthy mothers during the last eight weeks of pregnancy, and did not correlate with the outcome measures. However, because mothers with this disease complied with specific dietary recommendations only after they had been diagnosed with GDM, unhealthy maternal nutrition during early pregnancy may theoretically be one of the components contributing to abnormalities in the course of male minipuberty, and this possibility requires confirmation.

This study also shows for the first time that male descendants of women with poorly controlled disease were characterized by transiently reduced concentrations of DHEA-S, the dominant circulating form of dehydroepiandrosterone (DHEA) [59]. There are at least four explanations for between-group differences in salivary DHEA-S concentrations. Firstly, low DHEA-S levels may suggest mild adrenal failure. In line with this explanation, 11–13-year-old children born to women with GDM were reported to have lower salivary cortisol levels than their healthy peers [60]. Secondly, considering that low levels of DHEA and DHEA-S are regarded as general markers of poor health and an epiphenomenon of subclinical diseases [59], low DHEA-S levels may be a response to changes in the intrauterine environment caused by maternal hyperglycemia. Thirdly, low DHEA-S levels may result from impaired conversion of DHEA, or increased utilization of this hormone in other metabolic pathways. Lastly, we cannot exclude the impact of gestational hyperglycemia on the salivary pharmacokinetics of DHEA-S. From a theoretical point of view, low DHEA-S levels during the first six months of life may be undesirable owing to neuroprotective properties of DHEA and DHEA-S, and their putative role in the proper development and maturation of the brain [61].

The obtained results allow us to draw other conclusions. Despite differences in testosterone concentrations, there were no correlations between testosterone and androstenedione. This observation may support previous findings indicating that the adrenals are the main source of androstenedione in infant boys [62]. Secondly, considering abundant production and non-stressful collection of saliva, assessment of salivary testosterone in the first months of life may provide a window of opportunity to predict the course of puberty in male descendants of women with GDM. This conclusion is supported by the finding that early assessment of gonadotropins and testosterone predicted pubertal growth of the testes and penis in boys with hypogonadism [4]. Lastly, measurements of salivary testosterone and DHEA-S in infancy may serve as a retrospective marker of metabolic control of GDM, and may theoretically help to assess risk of long-term complications in the offspring.

The findings of this study have to be seen in light of some limitations. Due to the small number of participants per group (though exceeding the required sample size), they should be interpreted as hypothesis-generating, and warranting large-scale validation. Because no participant entered the study under 15 days of age, the study does not allow us to ascertain the earliest period (the first two weeks) of male puberty. Owing to the fact that some maternal data were collected retrospectively, partially based on information obtained from women during the first study visit, the results might have been affected by recall bias. Because all mothers had GDM, it cannot be ruled out that the course of minipuberty may be different in sons of women with type 1 and type 2 diabetes. Despite matching for a family history of diabetes during pregnancy, we cannot completely exclude the possibility that genetic factors might have influenced our findings. The study does not provide a mechanistic explanation for our findings. Immunoassays used in this study are susceptible to interference with compounds (endogenous steroids and their metabolites) having structural similarity to the target steroid of the assay [63] and may overestimate salivary hormone concentrations. Lastly, although the study design minimized the impact of random diurnal, seasonal and analytical variations in the measured hormones, the regression toward the mean effect cannot be completely ruled out [64].

**Figure 10:**
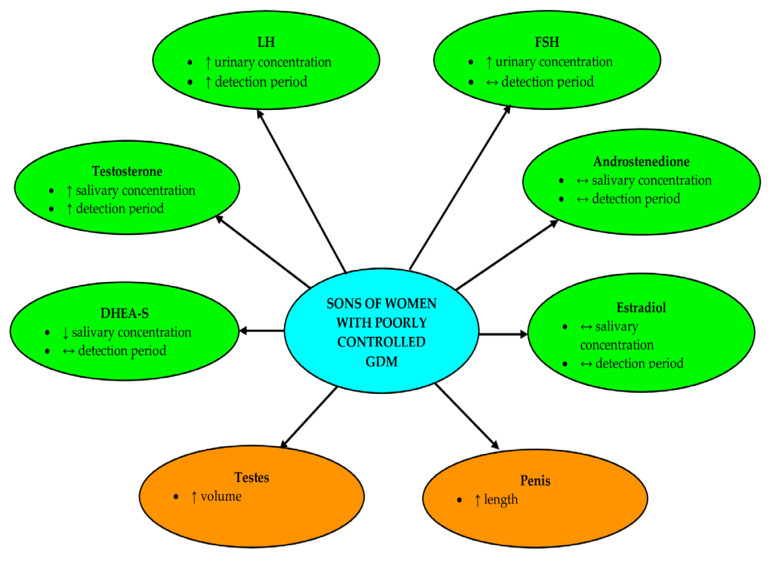
Measured hormones and sexual organs in infant sons of women with poorly controlled GDM.

Summing up, our study has shown that maternal GDM may affect concentrations of gonadotropins and steroid hormones, their detection periods and postnatal growth of male sexual organs in the first postnatal year (Figure 10). These changes were observed only if the disease was poorly controlled during pregnancy.

## 5. Conclusions

Sons of women with poorly controlled GDM are characterized by increased and longer-acting activation of the reproductive axis, and faster growth of male genital organs in infancy. The impact of GDM on the male reproductive system in this period of life likely depends on severity of hyperglycemia during pregnancy, and can be prevented by effective treatment. Poorly controlled GDM is also complicated by a transient decrease in salivary DHEA-S. However, GDM-induced activation of the hypothalamic–pituitary–testicular axis is not directly determined by calorie and nutrient intake. The obtained results suggest that metabolic changes associated with poorly controlled GDM may affect the course of male minipuberty. Given the potential practical significance of our findings, their novelty and the methodological limitations, the obtained results require confirmation in subsequent studies. Further research should also allow us to better understand molecular and cellular mechanisms of interaction between GDM and activation of the reproductive axis in infancy, as well as to investigate the course of minipuberty in infants born to mothers with the remaining types of diabetes.

## Figures and Tables

**Table 1:** Characteristics of mothers of infant boys who completed the study.

Variable	Group A	Group B	Group C	*p*-Value		
				A vs. B	A vs. C	B vs. C
Number (n)	29	28	29	-	-	-
Age (years)	35 ± 9	35 ± 9	36 ± 9	1.0000	0.6738	0.6766
University/secondary/primary or vocational education (%)	52/38/19	54/36/10	55/38/7	0.7365	0.6472	0.6894
Employment rate/white-collar/pink-collar/blue-collar workers (%)	90/48/34/7	93/54/29/10	93/52/31/10	0.7856	0.8015	0.8875
High/middle/low socioeconomic status (%)	14/59/27	11/64/25	14/66/21	0.7261	0.5664	0.6929
Smokers/non-smokers (n)	11/18	10/18	10/19	0.8623	0.7846	0.9224
BMI (kg/m^2^)	25.6 ± 4.7	25.3 ± 4.2	24.4 ± 4.5	0.8006	0.3249	0.4388
Systolic blood pressure (mmHg)	124 ± 17	118 ± 16	118 ± 19	0.1759	0.2103	1.0000
Diastolic blood pressure (mmHg)	83 ± 8	81 ± 7	80 ± 7	0.3202	0.1342	0.5919
Mean total daily calorie intake (kcal/kg)	31.7 ± 4.0	31.4 ± 3.5	32.7 ± 4.2			
Mean daily carbohydrate intake (g)	270 ± 43	259 ± 38	276 ± 50	0.3112	0.6261	0.1551
Mean daily lipid intake (g)	92 ± 12	89 ± 10	88 ± 14	0.3106	0.2477	0.7582
Mean daily protein intake (g)	93 ± 10	91 ± 9	88 ± 12	0.4314	0.1001	0.2916
Mean daily fiber intake (g)	35 ± 6	36 ± 6	34 ± 7	0.5317	0.5615	0.2526
Mean daily vitamin D intake during pregnancy (µg)	43.0 ± 13.1	44.9 ± 13.5	47.8 ± 14.9	0.5919	0.1980	0.4451
Mean fasting glucose during pregnancy (mmol/L)	5.50 ± 0.61	4.39 ± 0.50	4.36 ± 0.47	<**0.0001**	<**0.0001**	0.8163
Present/absent family history of diabetes during pregnancy (n)	10/49	10/51	11/53	0.9349	0.9720	0.9055

Unless otherwise stated, the data are presented as the mean ± standard deviation. BMI was calculated based on data from the first pregnancy visit during the first trimester. Mean daily intake of calories, macronutrients and vitamin D was calculated based on data from the last eight weeks of pregnancy. Statistical significance is marked in bold.

**Table 2:** Baseline characteristics of infant boys who completed the study.

Variable	Group A	Group B	Group C	*p*-Value		
				A vs. B	A vs. C	B vs. C
Number (n)	29	28	29	-	-	-
Gestational age of delivery (weeks)	39 ± 2	39 ± 2	40 ± 1	1.0000	0.0621	0.0644
Birth order: first/second/third and subsequent (%)	55/38/7	54/36/10	52/41/7	0.7864	0.6128	0.6815
Body length (cm)	54.8 ± 1.4	54.6 ± 1.9	54.4 ± 1.6	0.6519	0.3152	0.6687
Head circumference (cm)	37.6 ± 0.8	36.4 ± 0.9	37.8 ± 0.7			
Body weight (kg)	4.78 ± 0.60	4.43 ± 0.53	4.38 ± 0.55	**0.0234**	**0.0105**	0.7282
Weight-for-length percentile	82 ± 9	52 ± 23	61 ± 21	<**0.0001**	<**0.0001**	0.1284
Present/absent breastfeeding (n)	23/6	23/5	24/5	0.7865	0.7376	0.9512
Total daily vitamin D intake (µg)	13.2 ± 1.5	13.6 ± 1.8	12.9 ± 1.4	0.3654	0.4344	0.1163

Unless otherwise stated, the data are presented as the mean ± standard deviation. Statistical significance is marked in bold.

**Table 3:** The area under the curve for the assessed hormones and sexual organs.

Variable	Group A	Group B	Group C	*p*-Value		
				A vs. B	A vs. C	B vs. C
Salivary testosterone (pmol/L × months)	1523 ± 652	702 ± 322	718 ± 340	<**0.0001**	<**0.0001**	0.8560
Salivary androstenedione (pmol/L × months)	497 ± 275	531 ± 302	554 ± 209	0.3780	0.4555	0.7700
Salivary DHEA-S (pmol/L × months)	1497 ± 598	1858 ± 682	1828 ± 635	**0.0379**	**0.0457**	0.8591
Salivary estradiol (pmol/L × months)	42 ± 27	46 ± 29	47 ± 32	0.5918	0.5228	0.9030
Urinary LH (unit/mmol of creatinine × months)	17.98 ± 8.98	8.39 ± 3.95	8.25 ± 4.12	<**0.0001**	<**0.0001**	0.8964
Urinary FSH (unit/mmol of creatinine × months)	9.38 ± 3.60	6.51 ± 3.12	6.29 ± 3.05	**0.0022**	**0.0008**	0.7888
Testosterone volume (μL × months)	498 ± 56	438 ± 51	450 ± 49	**0.0001**	**0.0010**	0.3689
Penile length (mm × months)	525 ± 65	477 ± 61	467 ± 60	**0.0058**	**0.0008**	0.5352

The data are presented as the mean ± standard deviation. Values for the time points at which saliva and urine were not collected (months 7, 9 and 11) were extrapolated from the curve. Statistical significance is marked in bold.

**Table 4:** Correlations between the measured variables.

Correlated Variables		Group A	Group B	Group C
LH	Testosterone	0.46 [*p* = 0.0002]– 0.68 [*p* < 0.0001]	0.49 [*p* < 0.0001]– 0.70 [*p* < 0.0001]	0.50 [*p* < 0.0001]– 0.71 [*p* < 0.0001]
FSH	Testicular volume	0.26 [*p* = 0.0488]– 0.37 [*p* = 0.0152]	0.40 [*p* = 0.0014]– 0.60 [*p* < 0.0001]	0.39 [*p* = 0.0105]– 0.58 [*p* < 0.0001]
Testosterone	Penile length	0.34 [*p* = 0.0286]– 0.44 [*p* = 0.0004]	0.30 [*p* = 0.0465]– 0.42 [*p* = 0.0006]	0.31 [*p* = 0.0395]– 0.43 [*p* = 0.0005]
Mean maternal HbA_1c_	FSH	0.32 [*p* = 0.0302]– 0.46 [*p* = 0.0002]	0.10 [*p* = 0.3512]– 0.21 [*p* = 0. 0628]	not assessed
Mean maternal HbA_1c_	LH	0.30 [*p* = 0.0355]– 0.47 [*p* = 0.0001]	0.12 [*p* = 0.2865]– 0.22 [*p* = 0.0558]	not assessed
Mean maternal HbA_1c_	Testosterone	0.31 [*p* = 0.0322]– 0.43 [*p* = 0.0008]	0.08 [*p* = 0.6442]– 0.19 [*p* = 0.1020]	not assessed
Mean maternal HbA_1c_	DHEA-S	−0.34 [*p* = 0.0255]– −0.46 [*p* = 0.0002]	−0.04 [*p* = 0.7842]– −0.21 [*p* = 0.06551]	not assessed

The data represent the correlation coefficients (r values).

## Data Availability

The data that support the findings of this study are available from the corresponding author upon reasonable request. The data are not publicly available due to privacy and legal restrictions.

## Supplementary Materials

The following supporting information can be downloaded at https://www.mdpi.com/article/10.3390/nu16234145/s1, Table S1. Salivary testosterone concentrations in infant boys participating in the study; Table S2. Salivary androstenedione concentrations in infant boys participating in the study; Table S3. Salivary DHEA-S concentrations in infant boys participating in the study; Table S4. Salivary estradiol concentrations in infant boys participating in the study; Table S5. Urinary LH levels in infant boys participating in the study; Table S6. Urinary FSH levels in infant boys participating in the study; Table S7. Testicular volume in infant boys participating in the study; Table S8. Penile length in infant boys participating in the study.
