# Supplementary material for: The Impact of Maternal Gestational Diabetes Mellitus on Minipuberty in Boys
Source: Nutrients. 2024 Nov 29;16(23):4145. doi: 10.3390/nu16234145 (PMC11644001; doi:10.3390/nu16234145)
Supplement: Supplementary file 1 [file nutrients-16-04145-s001.zip › nutrients-3311176-supplementary.pdf]

Supplementary Table S1. Salivary testosterone concentrations in infant boys participating in the study.

Age	Group A	Group B	Group C
Month 1	185±60[d]	128±50	132±55
Month 2	206±58[d]	160±55[a]	173±60[a]
Month 3	203±75[d]	128±52[b]	130±59[b]
Month 4	188±73[d]	120±49[b]	118±52[b]
Month 5	198±69[d]	118±42[b]	112±48[b]
Month 6	190±82[d]	48±22[a,b,c]	53±29[a,b,c]
Month 8	172±79[d]	Below LOD	Below LOD

The data are expressed in pmol/L, and presented as the mean ± standard deviation. Testosterone was undetectable in saliva since month 10 to month 12 in group A, and since month 8 to month 12 in groups B and C. In statistical comparisons, limit of detection (LOD) value was assigned for testosterone in groups B and C at month 8. [a]$p<0.05$ *vs*. levels at month 1 in the same study group; [b]$p<0.05$ *vs*. levels at month 2 in the same study group; [c]$p<0.05$ *vs*. levels at months 3-5 in the same study group; [d]$p<0.05$ *vs*. levels in groups B and C at the same time point.

Supplementary Table S2. Salivary androstenedione concentrations in infant boys participating in the study.

Age	Group A	Group B	Group C
Month 1	88±40	95±58	100±59
Month 2	100±52	108±60	110±49
Month 3	105±55	112±58	118±59
Month 4	98±46	103±55	102±42
Month 5	58±32[a]	62±35[a]	69±40[a]
Month 6	48±30[a]	51±29[a]	55±23[a]

The data are expressed in pmol/L, and presented as the mean ± standard deviation. Androstenedione was undetectable in saliva since month 8 to month 12. [a]$p<0.05$ *vs*. levels during the first 6 months of life.

Supplementary Table S3. Salivary DHEA-S concentrations in infant boys participating in the study.

Age	Group A	Group B	Group C
Month 1	130±55[a]	175±78	169±71
Month 2	135±62[a]	180±85	176±68
Month 3	140±60[a]	185±70	180±64
Month 4	134±58[a]	172±68	169±51
Month 5	142±55[a]	178±71	182±80
Month 6	131±53[a]	165±67	171±64
Month 8	146±50	167±64	160±55
Month 10	134±49	155±65	148±58
Month 12	132±51	158±52	155±65

The data are expressed in pmol/L, and presented as the mean ± standard deviation. [a]$p<0.05$ *vs*. levels in groups B and C at the same time point.

Supplementary Table S4. Salivary estradiol concentrations in infant boys participating in the study.

Age	Group A	Group B	Group C
Month 1	16±9	18±9	17±10
Month 2	14±7	14±8	17±9
Month 3	12±6	14±7	13±7

The data are expressed in pmol/L, and presented as the mean ± standard deviation. Estradiol was undetectable in saliva since month 4 to month 12.

Supplementary Table S5. Urinary LH levels in infant boys participating in the study.

Age	Group A	Group B	Group C
Month 1	2.31±0.81[d]	1.50±0.60	1.48±0.68
Month 2	2.35±0.75[d]	1.89±0.62[a]	1.93±0.71[a]
Month 3	2.40±0.78[d]	1.52±0.58[b]	1.46±0.62[b]
Month 4	2.21±0.83[d]	1.48±0.68[b]	1.38±0.59[b]
Month 5	2.15±0.68[d]	1.35±0.59[b]	1.29±0.50[b]
Month 6	2.29±0.78[d]	0.65±0.35[a,b,c]	0.71±0.42[a,b,c]
Month 8	2.08±0.73[d]	Below LOD	Below LOD

The data are expressed in international units per mmol of creatinine, and presented as the mean ± standard deviation. LH was undetectable in saliva since month 10 to month 12 in group A, and since month 8 to month 12 in groups B and C. In statistical comparisons, limit of detection (LOD) value was assigned for LH in groups B and C at month 8. [a]$p<0.05$ *vs*. levels at month 1 in the same study group; [b]$p<0.05$ *vs*. levels at month 2 in the same study group; [c]$p<0.05$ *vs*. levels at months 3-5 in the same study group; [d]$p<0.05$ *vs*. levels in groups B and C at the same time point.

Supplementary Table S6. Urinary FSH levels in infant boys participating in the study.

Age	Group A	Group B	Group C
Month 1	1.28±0.40[b]	0.87±0.25	0.93±0.42
Month 2	1.34±0.37[b]	0.90±0.34	0.98±0.38
Month 3	1.25±0.46[b]	0.94±0.38	0.87±0.35
Month 4	1.38±0.48[b]	0.98±0.40	1.00±0.40
Month 5	1.22±0.38[b]	0.88±0.36	0.90±0.34
Month 6	1.20±0.35[b]	0.84±0.32	0.88±0.31
Month 8	0.74±0.38[a,b]	0.45±0.22[a]	0.49±0.24[a]

The data are expressed in international units per mmol of creatinine, and presented as the mean ± standard deviation. FSH was undetectable in urine since month 10 to month 12. [a]$p<0.05$ *vs*. levels during the first 6 months of life; [b]$p<0.05$ *vs*. levels in groups B and C at the same time point.

Supplementary Table S7. Testicular volume in infant boys participating in the study.

Age	Group A	Group B	Group C
Month 1	0.31±0.07	0.30±0.06	0.32±0.07
Month 2	0.34±0.07	0.32±0.07	0.34±0.07
Month 3	0.38±0.07[a]	0.37±0.06[a]	0.38±0.06[a]
Month 4	0.41±0.06[a,d]	0.37±0.06[a]	0.38±0.05[a]
Month 5	0.44±0.05[a,b,d]	0.40±0.06[a,b]	0.41±0.05[a,b]
Month 6	0.45±0.07[a,b,d]	0.40±0.06[a,b]	0.41±0.06[a,b]
Month 8	0.44±0.06[a,b,d]	0.37±0.05[a,c]	0.38±0.05[a,c]
Month 10	0.44±0.05[a,b,d]	0.37±0.05[a,c]	0.37±0.06[a,c]
Month 12	0.44±0.06[a,b,d]	0.36±0.06[a,c]	0.37±0.05[a,c]

The data are expressed in mL, and presented as the mean ± standard deviation. [a]$p<0.05$ *vs*. values at months 1-2 in the same study group; [b]$p<0.05$ *vs*. values at months 3-4 in the same study group; [c]$p<0.05$ *vs*. values at months 5-6 in the same study group; [d]$p<0.05$ *vs*. values in groups B and C at the same time point.

Supplementary Table S8. Penile length in infant boys participating in the study.

Age	Group A	Group B	Group C
Month 1	36±6	34±5	34±4
Month 2	38±5[c]	35±5	34±5
Month 3	41±5[a,c]	38±5[a]	37±4[a]
Month 4	42±5[a,c]	39±5[a]	38±5[a]
Month 5	45±5[a,b,c]	40±6[a]	39±6[a]
Month 6	45±5[,a,b,c]	40±7[a]	39±6[a]
Month 8	46±6[a,b,c]	42±5[a,b]	41±4[a,b,]
Month 10	46±5[a,b,c]	42±4[a,b]	41±5[a,b]
Month 12	47±6[a,b,c]	42±5[a,b]	42±6 [a,b]

The data are expressed in mm, and presented as the mean ± standard deviation. [a]$p<0.05$ *vs*. values at months 1-2 in the same study group; [b]$p<0.05$ *vs*. values at months 3-4 in the same study group; [c]$p<0.05$ *vs*. values in groups B and C at the same time point.
